# Familial Discoid Medial Meniscus Tear in Three Members of a Family: A Case Report and Review of Literature

**DOI:** 10.1155/2014/285675

**Published:** 2014-12-08

**Authors:** Raheel Ahmed Ali, Scott McKay

**Affiliations:** ^1^Baylor College of Medicine, Houston, TX 77030, USA; ^2^Department of Pediatric Orthopaedics, Texas Children's Hospital, Baylor College of Medicine, Houston, TX 77030, USA

## Abstract

*Background*. A discoid meniscus is a thickened variant of the normal C-shaped meniscus prone to injury. Discoid medial meniscal tears have rarely been reported within families and may suggest familial or developmental origins. *Methods*. We report the cases of two Caucasian brothers with symptomatic discoid medial meniscus tears. A literature review was conducted addressing discoid medial meniscus and cases of familial meniscus tears. *Case Presentation*. Physically active brothers presented with progressively worsening knee pain. MRI revealed medial meniscus tears in both brothers. The family history of medial meniscus tears in their mother and the discoid medial meniscus injuries found on arthroscopy suggested evidence for familial discoid medial meniscus tears. *Conclusions*. Discoid medial meniscus tears within a family have not been previously reported. Two cases of families with discoid lateral meniscus tears have been reported. Discoid medial meniscus is rare relative to the discoid lateral meniscus and predisposes children to symptomatic tears.

## 1. Introduction

Menisci are C-shaped cartilaginous padding between the tibial plateau and the femoral condyles composed of type-2 collagen arranged in a circumferential pattern. Menisci function to (1) distribute forces equally across the joint surface, (2) stabilize the contact between the femur and tibia, (3) aid in joint proprioception, and (4) aid in lubrication [1, 2]. Meniscal injuries are classified as a tear or degeneration. MRI produces a high-resolution image with 96% sensitivity and 97% specificity for detecting meniscal tears [1]. Meniscal degeneration appears on MRI as increased signal intensity that does not reach the articular surface while meniscal tears are linear hyperintense signals that reach the articular surface. Deficiency of the meniscus leads to unequal contact between the femur and tibia leading to osteoarthrosis. Removal of the meniscus in adolescents increases the risk of osteoarthritis by at least three times after thirty years from the initial injury [2].

A discoid meniscus is a thickened disk-like morphologic variant of a normal meniscus [3]. Discoid menisci can be partial or complete depending on the amount of tibial plateau they cover. They can also be categorized as Wrisberg variant, which has no posterior capsular attachment or tibial attachment, which precludes to instability and hypermobility [1]. Discoid menisci are prone to early tearing and degeneration. Of the 13 cases by Chen et al., 7 were complete discoid menisci and 6 were partial discoid menisci. Wrisberg type discoid medial meniscus has not been reported [3]. Occurrences of discoid medial meniscus are much less than those of discoid lateral menisci. One study found the incidence of discoid medial meniscus to be 0.12%, compared with 1.5% for lateral meniscus when investigating over 14,000 menisci. Bilateral cases are even rarer with an incidence of 0.012% [4].

We report the first reported case of familial partial discoid medial meniscus with two brothers and their mother who underwent arthroscopic confirmation of abnormally large medial menisci with early intrasubstance degeneration and subsequent tearing.

## 2. Case Report

**Figure 1:**
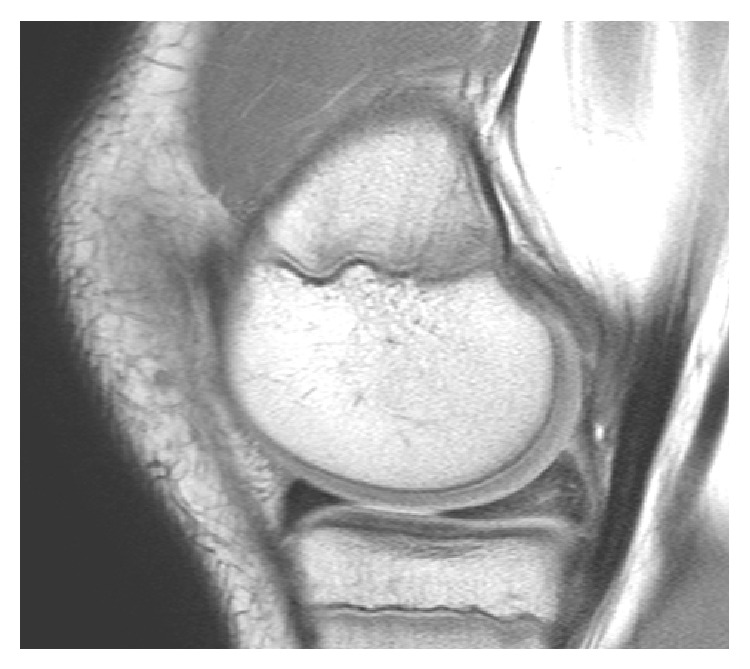
Sagittal MRI of the left medial meniscus with intrasubstance degeneration in the posterior horn.

A 14-year-old boy presented with nontraumatic bilateral knee pain. The patient reported more pain and popping sensations in the posteromedial aspect of his left knee. Eight weeks of physical therapy did not relieve his pain. Physical exam revealed no knee effusions, no ligamentous instability, and full range of motion. The McMurray test on the left knee resulted in pain along the medial joint line and a popping sensation. Left knee MRI revealed a large posterior horn medial meniscus with intrasubstance signal in the posterior horn likely representing degeneration (Figure 1). MRI of the right knee revealed an increased signal in the posterior horn with an enlarged medial meniscus. Due to the MRI findings and persistent symptoms, arthroscopic treatment was recommended.

**Figure 2:**
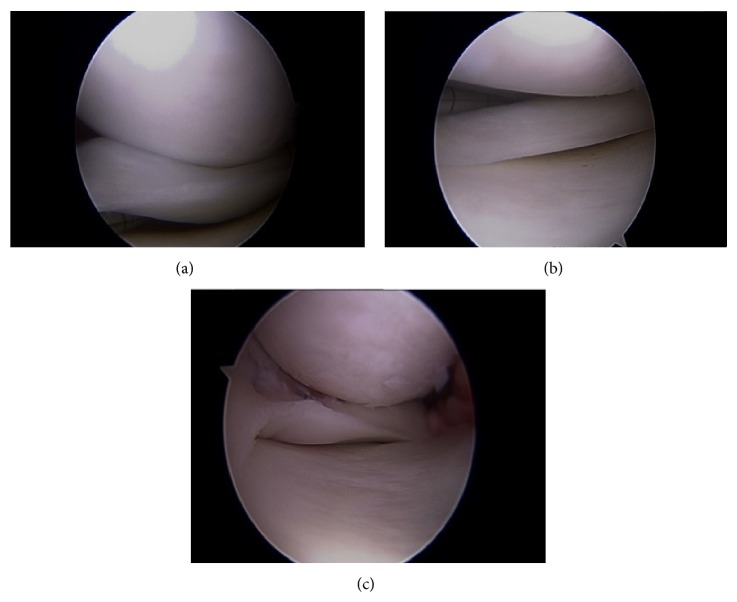
(a) Unusually large posterior horn suggesting partial discoid medial meniscus. (b) Probe inserted into the intrasubstance degeneration via the superior surface tear. (c) Medial meniscus after repair of peripheral fragment.

The patient underwent arthroscopic repair of the left medial meniscus without complications. A small peripheral tear in the superior surface of the medial meniscus allowed access to the intrasubstance degeneration (Figure 2(a)). The tear was repaired with two all-inside meniscal repair devices (Figures 2(b) and 2(c)). Its meniscocapsular attachments were normal. The patient was discharged in good condition on crutches with partial weight bearing. He returned to full sports contact after 4–6 months of physical therapy.

**Figure 3:**
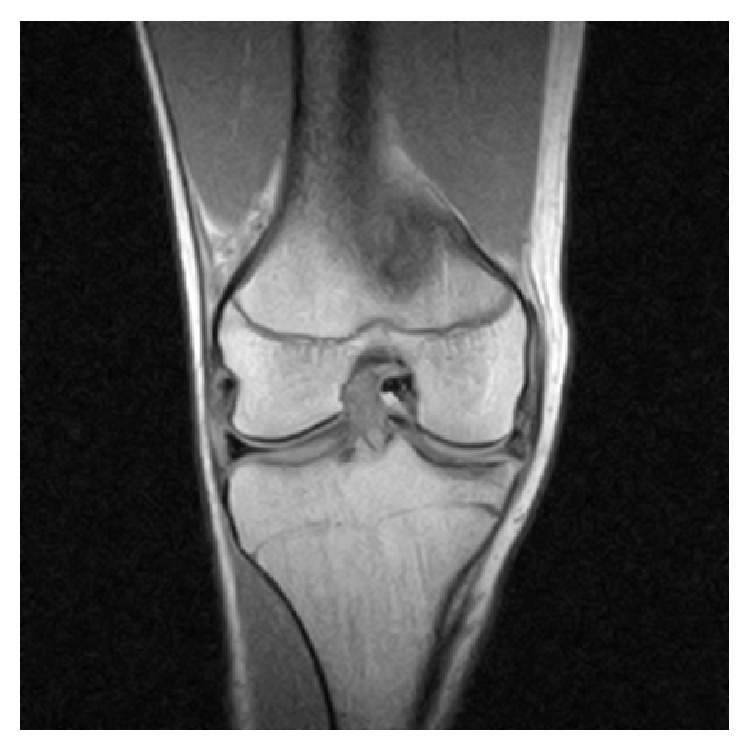
Coronal MRI of the right knee revealing intra-articular and bucket-handle medial meniscus tear with the displaced fragment located in the intercondylar notch.

**Figure 4:**
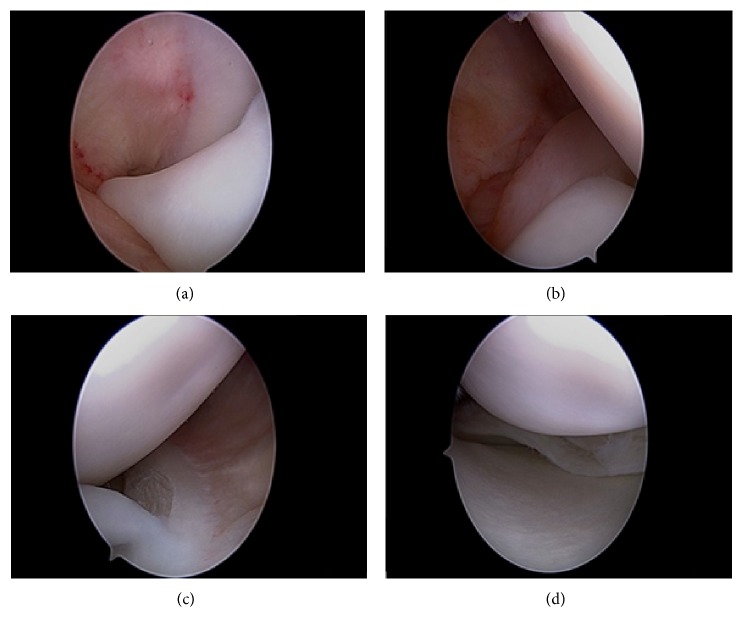
(a) Large displaced medial meniscus fragment located in the intercondylar notch. (b) Posterior edge of the tear. (c) Anterior edge of the tear. (d) Medial meniscus remaining after debridement.

A 16-year-old male, the above patient's brother, who is a professional dancer presented with 2 days of worsening right knee pain. He was treated for patellofemoral syndrome with three months of physical therapy. Audible sounds on flexion and extension of the knee were heard and full extension of the right knee was not possible. Physical exam revealed a large effusion. MRI of the right knee revealed a bucket-handle medial meniscus tear (Figure 3). Arthroscopy revealed a degenerative, complex horizontal discoid medial bucket-handle meniscus tear (Figures 4(a)–4(d)). The displaced meniscus fragment was removed. The remaining meniscus was larger than expected. The patient was discharged in good condition. After two months of physical therapy, the patient was able to return to preinjury activities.

**Figure 5:**
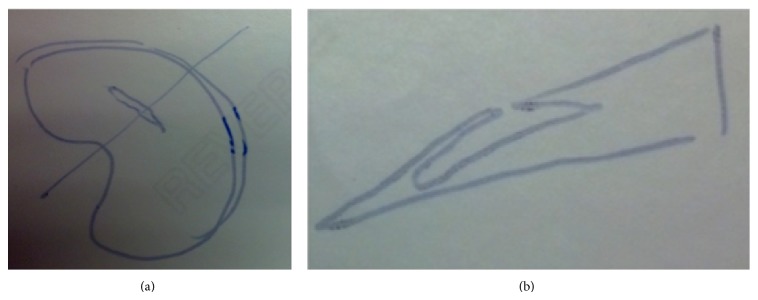
(a) Axial view diagram drawing displaying the mother's superficial tear. (b) Sagittal view diagram drawing of the mother's horizontal meniscal tear.

The mother of these two brothers reported she also had arthroscopic treatment of a medial meniscus tear. She shared with us a hand-written diagram given to her by the surgeon (Figures 5(a) and 5(b)). Her findings included a superior surface tear of the medial meniscus leading into an area of intrasubstance degeneration.

## 3. Discussion

Discoid menisci are rare. The origin of discoid menisci is unknown and debated. Smillie et al. theorized that discoid menisci occur due to persistence of the embryonic disk-shaped menisci arrested in development. Kaplan et al. suggested discoid lateral menisci develop due to abnormal motion caused by absence of posterior tibial attachment. As discoid meniscus is a structural aberration of development, a genetic abnormality could possibly be at fault. No theories of genetic etiology for discoid meniscus have been brought forward to date.

Discoid meniscus tears often present in adolescents. We report 3 members of a Caucasian family with abnormally large-shaped medial menisci that presented with early degenerative changes. Just as discoid lateral meniscus is known to have intrasubstance degeneration and can be partially discoid in appearance, we propose that our 3 cases represent partial discoid medial meniscus. This would be the first report of familial discoid medial meniscus. Familial cases of lateral discoid meniscus tears have only been reported in two other instances to our knowledge. Cases of bilateral symptomatic discoid lateral meniscus tears in three brothers and one sister with unaffected parents were reported in France [5]. A second report of a father and two offspring with discoid lateral meniscus tears was also reported [6]. Discoid medial meniscus is quite uncommon. No familial cases of discoid medial meniscus are reported, but isolated cases have also been reported (Table 1). A literature review reveals discoid medial menisci detected in all ages, bilateral and unilateral cases, and complete or partial discoid meniscus cases.

In summary, we present a mother and two sons with symptomatic partial discoid medial meniscus with degenerative tears treated surgically. This is the first report of familial medial discoid meniscus. Further investigation into the pathophysiology of discoid meniscus development is required to determine a mechanism of this finding.

## Figures and Tables

**Table 1:** Reports of discoid medial meniscus since 2001.

Authors	Date	Number of cases	Age (years)	Arthroscopic findings
Choi et al. [7]	2001	2	18 and 31 years	Incomplete discoid meniscus on the medial and lateral sides

Yáñez-Acevedo [8]	2001	1	11 years	Bilateral discoid lateral meniscus and unilateral discoid medial meniscus with complete medial meniscus tear

Tachibana et al. [9]	2003	4	59, 33, 51, and 39 years	(i) Bilateral discoid medial meniscus and unilateral discoid lateral meniscus (ii) Unilateral medial and lateral discoid meniscus (iii) Two cases of bilateral medial and lateral discoid meniscus

Kim and Seo [10]	2006	1	22 years	Complete discoid medial meniscus in one knee and partial discoid medial meniscus in the other

Lee et al. [11]	2007	3	Unknown	Bilateral discoid medial menisci with abnormal attachment to the ACL

Kim and Lubis [12]	2010	1	44 years	Bilateral medial and lateral discoid menisci with anomalous insertion to the ACL

Flouzat-Lachaniette et al. [4]	2011	4	13–28 years	Partial discoid medial meniscus tears (2 horizontal tears, 1 anterior tear, and 1 vertical tear)

Cho [13]	2011	1	Unknown	Bilateral discoid medial meniscus with attachment to the ACL

Chen et al. [3]	2013	13	11–53 years	(i) Seven complete discoid medial menisci, 6 partial discoid medial menisci, 0 Wrisberg variants, 3 bucket-handle tears, 4 vertical tears, 4 horizontal tears, and 2 complex tears

Zhang et al. [14]	2013	1	27 years	Partial discoid medial meniscus tear with a complex horizontal tear

Chen et al. [15]	2012	1	13 years	Partial discoid medial meniscus tear with horizontal cleavage tear
